# Overexpressed pseudogene MT1L associated with tumor immune infiltrates and indicates a worse prognosis in BLCA

**DOI:** 10.1186/s12957-021-02231-4

**Published:** 2021-04-22

**Authors:** Yanpeng Ding, Nuomin Liu, Mengge Chen, Yulian Xu, Sha Fang, Wenbin Xiang, Xinying Hua, Gaili Chen, Yahua Zhong, Haijun Yu

**Affiliations:** 1 Department of Radiation and Medical Oncology, Hubei Key Laboratory of Tumor Biological Behaviors, Hubei Cancer Clinical Study Center, Zhongnan Hospital of Wuhan University, Wuhan, 430071 China; 2 Department of Oncology, First People’s Hospital of Zaoyang, Zaoyang, 441200 China; 3 Wuhan University, Wuhan, 430071 Hubei Province China

**Keywords:** Pseudogene, Metallothionein, Urinary bladder neoplasms, Tumor microenvironment, Prognosis

## Abstract

**Background:**

BLCA is a common cancer worldwide, and it is both aggressive and fatal. Immunotherapy (ICT) has achieved an excellent curative effect in BLCA; however, only some BLCA patients can benefit from ICT. MT1L is a pseudogene, and a previous study suggested that MT1L can be used as an indicator of prognosis in colorectal cancer. However, the role of MT1L in BLCA has not yet been determined.

**Methods:**

Data were collected from TCGA, and logistic regression, Kaplan-Meier plotter, and multivariate Cox analysis were performed to demonstrate the correlation between the pseudogene MT1L and the prognosis of BLCA. To identify the association of MT1L with tumor-infiltrating immune cells, TIMER and TISIDB were utilized. Additionally, GSEA was performed to elucidate the potential biological function.

**Results:**

The expression of MT1L was decreased in BLCA. Additionally, MT1L was positively correlated with immune cells, such as Tregs (*ρ* = 0.708) and MDSCs (*ρ* = 0.664). We also confirmed that MT1L is related to typical markers of immune cells, such as PD-1 and CTLA-4. In addition, a high MT1L expression level was associated with the advanced T and N and high grade in BLCA. Increased expression of MT1L was significantly associated with shorter OS times of BLCA patients (*p* < 0.05). Multivariate Cox analysis revealed that MT1L expression could be an independent prognostic factor in BLCA.

**Conclusion:**

Collectively, our findings demonstrated that the pseudogene MT1L regulates the immune microenvironment, correlates with poor survival, and is an independent prognostic biomarker in BLCA.

## Background

Bladder cancer, a heterogeneous disease, is the second most prevalent cancer involving the urinary system, with approximately 430,000 newly diagnosed cases worldwide [1]. Patients with superficial tumors, which account for approximately 70% of bladder cancers, can be treated with transurethral resection of the bladder (TURB), though generally not life-threatening but have higher risks of recurrence [2]. In contrast, patients accompanying muscle invasion have poor prognoses and higher distant metastasis rates [3]. Cisplatin-based systemic chemotherapy remains the first-line treatment for bladder cancer patients with advanced disease and metastasis [4], although long-term responses are rare, and the recurrence rate is not improving. In addition, half of MIBC patients are cisplatin-ineligible, and there is no effective standard treatment for these patients [5]. The underlying molecular mechanism of BLCA remains unclear. It is necessary and meaningful to explore the mechanisms of this disease and the factors affecting its prognosis.

MT1L belongs to the family of metallothioneins (MTs) [6]. Evidence has shown that MTs can regulate cell growth and proliferation and protect the body against negative effects of oxidative stress, antineoplastic drugs, and radiation by binding heavy metals, such as zinc and copper [7–9]. There are four subtypes of MT proteins, MT-1, MT-2, MT-3, and MT-4 that are encoded by a single gene in humans. MT-1A, MT-1B, MT-1E, MT-1F, MT-1G, MT-1H, MT-1M, and MT-1x are active genes of the MT-1 subtype. The remaining MT-1 genes (MT-1C, MT-1D, MT-1I, MT-1J, and MT-1L) are pseudogenes [10]. Pseudogenes, abundant in the human genome, are a class of IncRNAs that control the expression of their homologous protein-coding genes and do not encode functional proteins due to different types of mutations in their coding sequence [11]. Recently, accumulating evidence has shown that pseudogenes play a crucial role in various diseases, especially in human cancers [12]. Several tumor-related pseudogenes have been proven to be indicators of human cancers. For instance, a pseudogene named DUXAP10 is upregulated in various cancers. It can promote HCC cell proliferation by activating the PI3K/AKT pathway and can be regarded as an independent prognostic biomarker in HCC [13, 14]. In addition, some pseudogenes have also been proven to contribute to the progression of several cancers, such as breast cancer, gastric cancer, and gallbladder carcinoma [15–17]. Therefore, the vital functions of pseudogenes in human cancers cannot be ignored.

In the available studies, we first identified a new pseudogene MT1L, of which the expression level, prognosis, and immune-associated of MT1L in BLCA have not been elucidated. Data were collected from TCGA. TIMER and TISIDB were used to demonstrate the association of MT1L with tumor-infiltrating immune cells. In addition, analyses with R-4.0.2, GEPIA, Kaplan-Meier plotter, UALCAN, and GSEA methods verified the expression of MT1L in human cancers and demonstrated the role of MT1L in BLCA. Eventually, the results identified both the strong correlation between MT1L and the tumor immune system and the essential prognostic value of MT1L in BLCA.

## Material and methods

### Data acquisition

Clinical follow-up information and RNA-seq data from 408 BLCA patients were screened out from TCGA [18]; patients with insufficient or missing data on overall survival time, TNM stage, and lymph node metastasis were excluded.

### GEPIA

Gene Expression Profiling Interactive Analysis (GEPIA), a web server, analyzes gene expression profiles and interaction in cancer and normal tissues based on TCGA and GTEx data [19]. To confirm whether the expression levels of MT1L differ between tumor tissues and the corresponding normal tissues in different types of human cancer, we used GEPIA to conduct differential expression analyses. Four-way analysis of variance (ANOVA) was applied to evaluate the differential expression using sex, age, ethnicity, and disease state (tumor or normal) as variables: Gene expression ~ sex + age + ethnicity + disease state. The Benjamini and Hochberg false discovery rate (FDR) method was used to adjust the *p*-values. Values of *p* < 0.05 indicated statistically significant differences (http://gepia.cancer-pku.cn/).

### TIMER analysis

Based on the RNA-seq data, we analyzed the TCGA database via Tumor Immune Estimation Resource site (TIMER) to reconfirm the expression of MT1L between tumor tissues and the corresponding normal tissues in different types of human cancer. The Wilcoxon test was used to calculate the statistical significance (*p-value < 0.05; **p-value <0.01; ***p-value <0.001). After that, TIMER was applied to determine the correlation between MT1L expression and immune infiltration in BLCA, using a previously published statistical deconvolution method [20] to infer the abundance of tumor-infiltrating immune cells (TIICs) from gene expression profiles [21]. In addition, we used the correlation module to explore the relationship between MT1L expression and typical immune cell markers, together with Spearman correlation analysis and the estimated statistical significance. *p*-values < 0.05 were considered statistically significant (https://cistrome.shinyapps.io/timer/).

### TISIDB analysis

Tumor-immune system interactions and drug bank database (TISIDB), an integrated repository portal for tumor-immune system interactions, combines five types of data resources to annotate each gene via ten kinds of analysis [22]. TISIDB uses the gene set variation analysis (GSVA) package and Spearman correlation analysis to infer the relative abundance of TILs and immunomodulators. In this study, we used TISIDB to infer the relations between the expression of MT1L and the abundance of immune cells and immunomodulators in BLCA, with *ρ* > 0.1 set as the criterion for identifying an influence on the immune system by MT1L expression (http://cis.hku.hk/TISIDB).

### UALCAN analysis

To obtain a survival curve for patients with different cancers, we first consulted the UALCAN database, a comprehensive and interactive web tool [23]. Student’s *t*-test was employed, and a *p*-value < 0.05 was considered to indicate statistical significance (http://ualcan.path.uab.edu).

### Kaplan-Meier plotter

Kaplan-Meier plotter, a web tool for assessing biomarkers, was used to analyze the association of MT1L expression with the overall survival (OS) of BLCA patients. This web tool can be used to evaluate the functions of 54,675 genes in 18,674 tumor samples, including samples of breast cancer, ovarian cancer, lung cancer, and gastric cancer [24]. The patient samples were classified into high- and low/moderate-expression groups to assess the prognostic value of MT1L, and the survival rate was calculated by the formula given below: St = (number of subjects alive at the start of the observation period − number of subjects who died)/number of subjects alive at the beginning of the observation period [25] (www.kmplot.com).

### Gene set enrichment analysis

Gene set enrichment analysis (GSEA) is a powerful analytical method to predict whether a gene set is enriched in a specific biological state [26]. The three key elements of GSEA are as follows: calculation of an enrichment score, estimation of the significance level of the ES, and adjustment for multiple hypothesis testing. More details about this method can be found in Aravind Subramanian’s study [26]. In our study, GSEA was performed to reveal the critical biological process and relevant signaling pathways affected by the expression level of MT1L. The cutoff criteria were *p* < 0.05 and normalized enrichment score (NES) > 1.5.

### Statistical analysis with R-4.0.2

Clinical information was obtained from TCGA and was analyzed by R-4.0.2. Using logistic regression and multivariate Cox regression analyses, we analyzed the correlations between clinicopathological characteristics and MT1L expression levels and evaluated the prognostic value of MT1L in BLCA, respectively. In addition, a correlation heatmap was generated for the correlation between MT1L expression and the infiltration of 22 types of immune cells. A *p*-value < 0.05 was considered statistically significant in this study.

## Results

### mRNA expression levels in BLCA and other human cancers

**Fig. 1:**
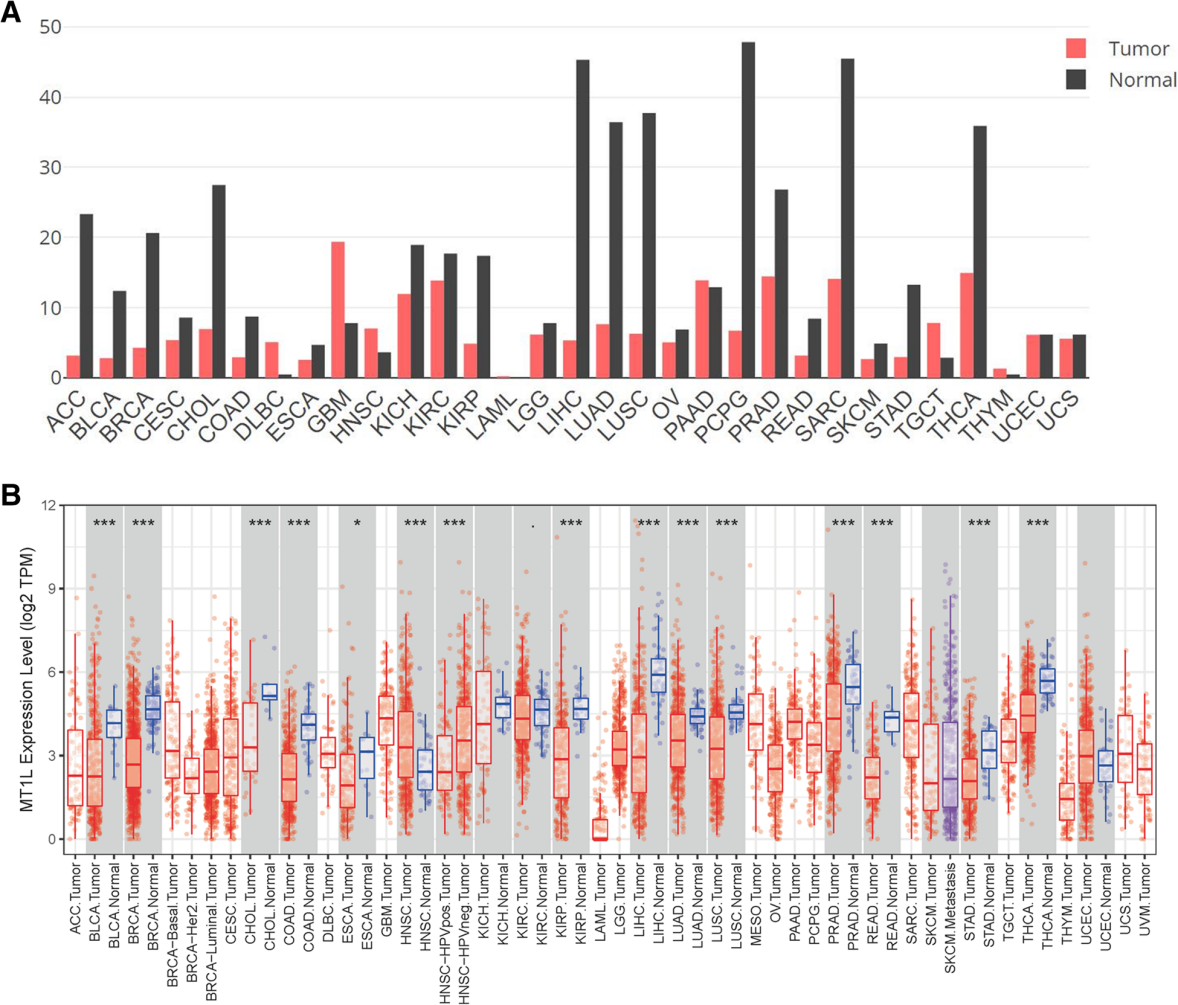
Analysis of MT1L expression in different tumor tissues. **a** MTL1 mRNA levels in 31 types of human cancers analyzed by GEPIA. **b** MT1L mRNA levels analyzed by TIMER (*p<0.05, **p<0.01, ***p<0.001)

Data on gene expression of 31 types in human cancers were acquired from GEPIA and TIMER. The analysis results are shown in Fig. 1a, b. Both databases showed consistent result patterns, with low MT1L expression in BLCA, BRCA, CHOL, COAD, KIRP, LIHC, LUAD, LUSC, READ, STAD, and THCA. Eventually, MT1L was identified to have low expression in BLCA.

### Strong correlation between MT1L and tumor immune infiltration in BLCA

**Fig. 2:**
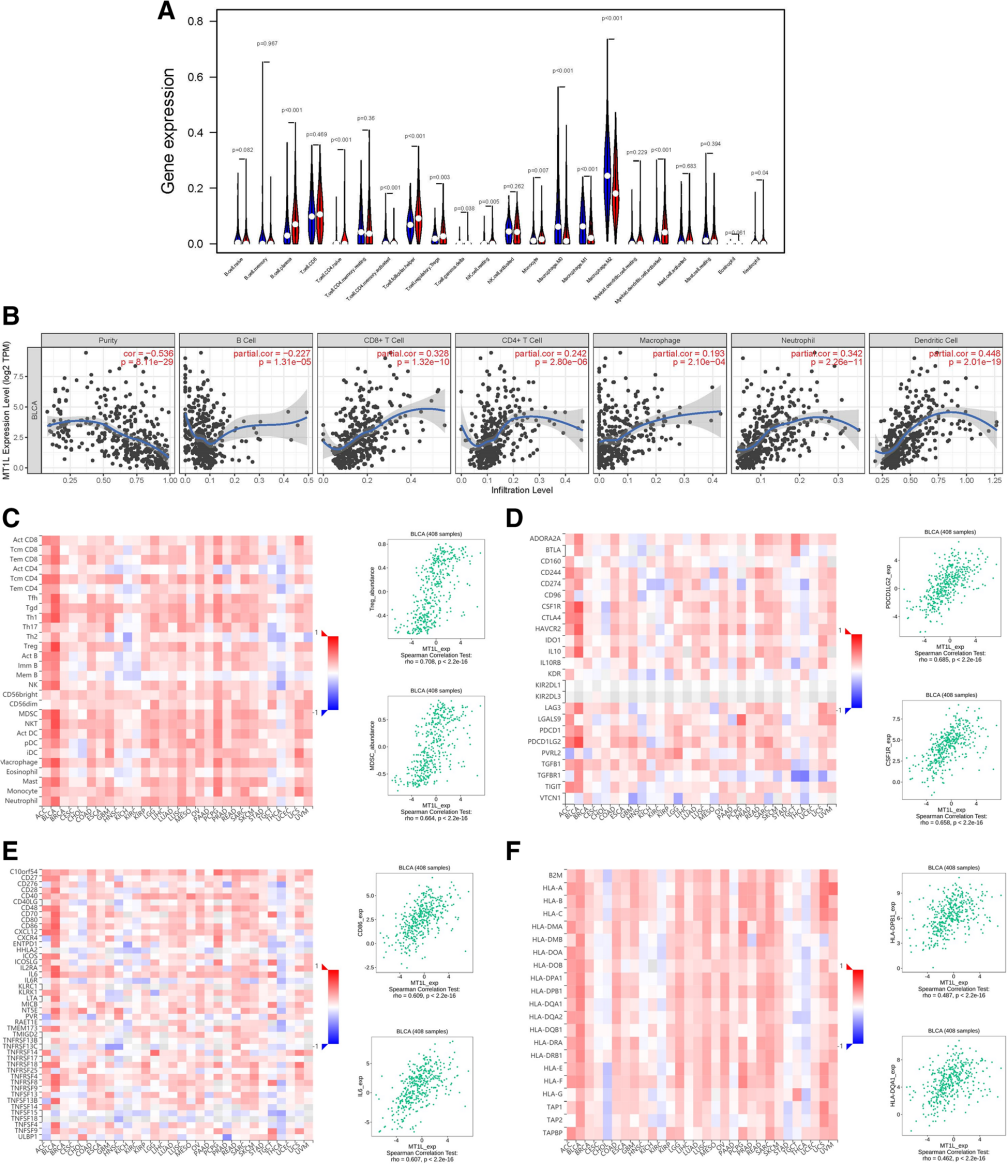
MT1L expression was related to immune infiltration. **a** Correlations between MT1L expression and the infiltration of 22 types of immune cells were assessed with R version 4.0.2 (*p*<0.001). **b** Correlations between MT1L expression and immune cells were analyzed by TIMER. **c**–**f** Relations between the abundances of TILs (**c**), immunoinhibitors (**d**), immunostimulators (**e**), and MHC molecules (**f**); the top 2 are displayed

Independent tumor-infiltrating lymphocytes are essential in the prediction of the overall survival rate. Thus, we used R-4.0.2 to analyze the correlation between MT1L expression and the infiltration of 22 types of immune cells based on 408 samples downloaded from the TCGA database. As shown in Fig. 2a, plasma B cells, naive CD4+ T cells, activated CD4+ memory T cells, T follicular helper cells, regulatory T cells, gamma delta T cells, resting NK cells, monocytes, M0 macrophages, M1 macrophages, M2 macrophages, activated myeloid dendritic cells, and neutrophil were the primary immune cells affected by MT1L expression.

TIMER was used to further identify the correlation between MT1L and immune infiltration in BLCA. Dendritic cells (*r* = 0.448, *p* < 0.001) had a certain correlation with MT1L expression. The results also showed associations between neutrophil (*r* = 0.342, *p* < 0.001), CD8+ T cells (*r* = 0.328, *p* < 0.001), CD4+ T cells (*r* = 0.242, *p* < 0.001), and MT1L expression (Fig. 2b).

The TISIDB database was used to evaluate the Spearman correlations between MT1L expression and the abundances of lymphocytes and immunomodulators (Fig. 2c–f). The results showed that MT1L expression was related to TILs. For example, regulatory T cells (Tregs) (*ρ*=0.708, *p*<2.2e−16), natural killer T cells (NKTs) (*ρ*=0.683, *p*<2.2e−16), myeloid-derived suppressor cells (MDSCs) (*ρ*=0.664, *p*<2.2e−16), T follicular helper cells (Tfhs) (*ρ*=0.619, *p*<2.2e−16), and effector memory CD8+ T cells (Tem-CD8) (*ρ*=0.549, *p*<2.2e−16) showed a strong correlation with MT1L expression. Furthermore, it is obvious that the correlation between MT1L expression and TIL abundances was stronger in BLCA than in other human cancers. Immunomodulators can be further classified into immunoinhibitors, immunostimulators, and MHC molecules. Regarding immunoinhibitors, MT1L expression exhibited a positive correlation with the expression of CSF1R, HAVCR2, IL10, PDCD1, and PDCD1LG2. Regarding immunostimulators, MT1L expression was correlated with the expression of CD28, CD70, CD86, IL2RA, IL6, TNFRSF4, and TNFRSF8. Regarding MHC molecules, the expression of HLA-B, HLA-DMB, HLA-DPB1, HLA-DQA1, HLA-DQB1, and HLA-DRB1 was related to MT1L expression.

Therefore, the results from all three databases show that MT1L is an essential and unique factor in the tumor immune microenvironment, especially in BLCA.

### Correlation between MT1L and immune cell type markers

The relationship between MT1L expression and typical immune cell markers was explored through the TIMER database (Table 1). We found a significant positive correlation of MT1L with CD8+ T cell markers, T cell (general) markers (CD3E, CD2), monocyte marker (CD86), natural killer cell markers (KIR2DL3, KIRIDL4), dendritic cell markers (HLA-DPB1, HLA-DQB1, HLA-DPA1), and markers of functional T cells, including Th1 marker (IFNG, TNF), a Treg marker (ENTPD1), and markers of T cell exhaustion (PD-1, CTLA4). Notably, we found that MT1L exhibited a definite relationship with markers of monocytes and M2 macrophages, which means that MT1L may regulate macrophage polarization in BLCA.

**Table 1:** The relationship between MT1L expression and typical markers of immune cells was analyzed with the TIMER database (*p<0.05, **p<0.01, ***p<0.001)

		BLCA			
		None		Purity	
Description	Gene markers	Cor	*p*	Cor	*p*
CD8+T cell	CD8A	0.447	***	0.227	***
	CD8B	0.395	***	0.249	***
T cell (general)	CD3D	0.438	***	0.185	**
	CD3E	0.487	***	0.215	***
	CD2	0.479	***	0.214	***
B cell	CD19	0.331	***	0.078	.14
	CD79A	0.381	***	0.106	*
Monocyte	CD86	0.622	***	0.418	***
	CD115(CSF1R)	0.674	***	0.494	***
TAM	CCL2	0.558	***	0.347	***
	CD68	0.431	***	0.251	***
	IL10	0.602	***	0.431	***
M1 macrophage	INOS(NOS2)	0.106	**	0.055	.30
	COX2(PTGS2)	0.134	**	0.026	.62
M2 macrophage	CD163	0.654	***	0.461	***
	VSIG4	0.674	***	0.505	***
	MS4A4A	0.648	***	0.455	***
Neutrophils	CD11b(ITGAM)	0.591	***	0.364	***
	CD66b(CEACAM8)	0.008	.87	0.036	.49
Natural killer cell	KIR2DL1	0.226	***	0.071	.18
	KIR2DL3	0.393	***	0.248	***
	KIR2DL4	0.375	***	0.222	***
	KIR3DL1	0.281	***	0.169	*
	KIR3DL2	0.282	***	0.132	*
	KIR3DL3	0.111	*	0.062	.24
	KIR2DS4	0.273	***	0.133	*
Dendritic cell	HLA-DPB1	0.497	***	0.235	***
	HLA-DQB1	0.472	***	0.243	***
	HLA-DPA1	0.450	***	0.207	***
	BDCA-4(NRP1)	0.541	***	0.456	***
	CD11c(ITGAX)	0.620	***	0.392	***
Th1	T-bet(TBX21)	0.436	***	0.190	**
	STAT4	0.565	***	0.361	***
	STAT1	0.366	***	0.187	**
	IFN-γ(IFNG)	0.393	***	0.219	***
	TNF-α(TNF)	0.384	***	0.244	***
Th2	GATA3	−0.489	***	−0.402	***
	STAT6	−0.263	***	−0.215	***
	STAT5A	0.237	***	0.034	.52
	IL13	0.202	***	0.036	.49
Treg	FOXP3	0.214	***	0.433	***
	IL2RA	0.606	***	0.395	***
	CD39(ENTPD1)	0.457	***	0.247	***
	TGFβ(TGFB1)	0.444	***	0.375	***
T cell exhaustion	PD-1(PDCD1)	0.475	***	0.248	***
	CTLA4	0.511	***	0.294	***
	LAG3	0.519	***	0.332	***
	TIM-3(HAVCR2)	0.621	***	0.410	***
	GZMB	0.533	***	0.328	***

Of note were the striking correlations between MT1L and dendritic cell markers (NRP1, ITGAX). It has been proven that DCs can promote tumor metastasis by increasing the abundance of Treg cells and reducing CD8^+^ T cell cytotoxicity [27]. In addition, the higher degree of association of MT1L expression with markers of T cell exhaustion and Tregs, such as LAG3, TIM-3, GZMB, FOXP3, IL2RA, and TGFB1, cannot be ignored. Studies show that FOXP3, a vital factor, restrains cytotoxic T cells from attacking tumor cells [28]. Additionally, the correlations between MT1L and T cell exhaustion markers mean that high expression of MT1L in BLCA leads to exhaustion of T cells. Therefore, the above results confirm the critical role of MT1L in immune escape in the bladder cancer microenvironment.

### Correlations between MT1L expression and clinicopathological characteristics

Given that the strong correlation between immune infiltration and MT1L was shown above, while the expression level and clinical value of MT1L in BLCA were not yet characterized, we used GEPIA and R version 4.0.2 to analyze the MT1L expression level and its correlation between clinicopathological characteristics in BLCA based on the data downloaded from the TCGA database. The results in Fig. 1 reveal that MT1L expression in BLCA tumor samples was lower than that in normal samples. Then, we evaluated the correlation between MT1L expression and several clinical characteristics of BLCA patients. A total of 408 BLCA samples from the TCGA database were included in our analysis. Using logistic regression, we found that MT1L has excellent diagnostic value in BLCA patients. The MT1L expression level significantly correlated with patient age (*p*=0.039), weight (*p*=0.01), race (Asian vs other, *p*=0.043; Asian vs White, *p*<0.001), N stage (N0 vs >N0, *p*=0.026), cancer stage (I–II vs III, *p*<0.001; I–II vs IV, *p*<0.001), T stage (<T2 vs T3, *p*<0.001; <T2 vs T4, *p*=0.001), and differentiation grade (high vs low, *p*=0.003), as shown in Table 2.

**Table 2:** The relationship between MT1L expression and clinical characteristics in BLCA was analyzed by logistic regression with 408 samples obtained from TCGA

Clinical characteristics	Number	OR	***p***-value
**Age**	408	1.02 (1.00–1.04)	0.039
**Gender**	408	0.76 (0.48–1.18)	0.216
**Weight**	408	1.01 (1.00–1.02)	0.01
**Race**			
**Asian vs others**	67	3.01 (1.06–9.21)	0.043
**Asian vs White**	369	5.56 (2.63–13.20)	<0.001
**Others vs White**	348	0.80 (0.33–1.87)	0.605
**M (M0 vs M1)**	207	2.95 (0.83–13.80)	0.118
**N (N0 vs >N0)**	366	0.61 (0.40–0.94)	0.026
**Stage**			
**I–II vs III**	272	2.53 (1.56–4.14)	0
**I–II vs IV**	266	2.50 (1.54–4.12)	0
**III vs IV**	274	1.06 (0.66–1.70)	0.81
**T**			
**<T2 vs T3**	317	2.26 (1.43–3.60)	0
**<T2 vs T4**	181	3.03 (1.59–5.98)	0.001
**T3 vs T4**	252	1.11 (0.62–2.01)	0.728
**Grade (high vs low)**	405	0.153 (0.04–0.46)	0.003

### The clinical value of MT1L in BLCA

**Fig. 3:**
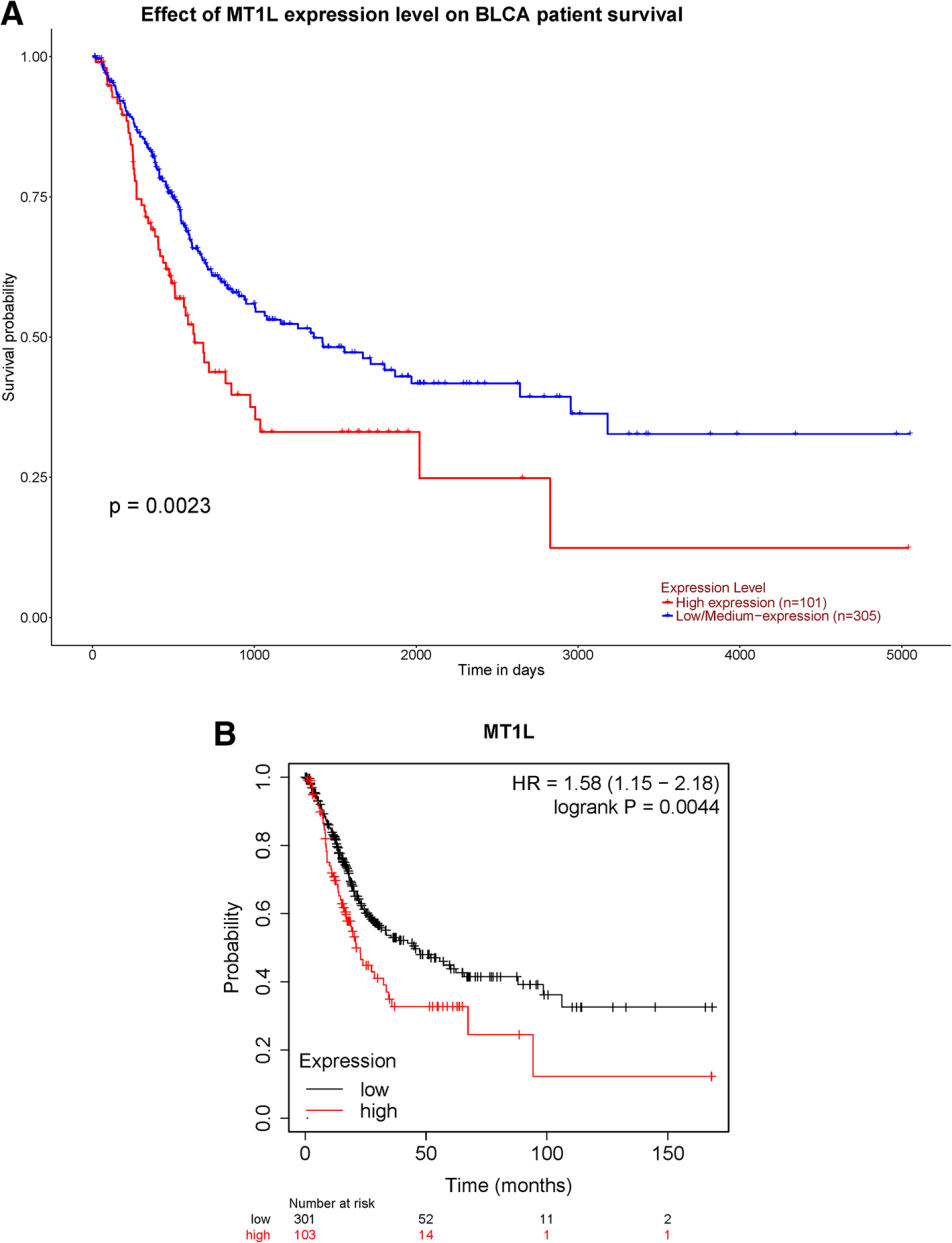
The survival curve obtained from **a** the UALCAN database and **b** Kaplan-Meier plotter. The red line indicates a high expression level of MT1L, and the other line indicates a low expression level

We consulted the UALCAN database and obtained the survival curves for patients with BLCA (Fig. 3a). The results revealed that higher expression of MT1L in BLCA indicated a shorter overall survival time among 406 BLCA patients (*n*=406, *p*=0.0023).

Kaplan-Meier plotter was used to further confirm the correlation between MT1L expression and OS (Fig. 3b). As the pictures show, increased expression of MT1L in BLCA indicated a shorter overall survival time among 404 patients (*n*=404, HR=1.58, 95% CI=1.15–2.18, *p*=0.0044). Similarly, high expression of MT1L in bladder carcinoma predicted an unfavorable prognosis.

Based on the correlation between MT1L expression and prognosis, we then conducted a multivariate Cox analysis. In this analysis, MT1L exhibited significant prognostic value in BLCA. Tumor stage, Person neoplasm status, and MT1L expression level were verified to be independent prognostic factors (Table 3).

**Table 3:** Multivariate Cox regression analysis of MT1L expression and other clinical characteristics

Clinical characteristics	HR (95% CI)	*p*-value
Gender	1.288 (0.861–1.926)	0.218
Weight	0.999 (0.990–1.228)	0.875
T	1.411 (1.080–1.843)	0.012*
Grade	0.768 (0.177–3.324)	0.724
Person neoplasm status	3.814 (2.640–5.509)	9.51E−13***
MTIL	1.087 (1.001–1.180)	0.048

### Gene sets enriched in BLCA with respect to the MT1L expression phenotype

GSEA is a powerful analytical method to predict whether a gene set is enriched in a specific biological state [26] and was performed to identify the key biological processes and relevant signaling pathways affected by the expression of MT1L (Table 4). GO enrichment analysis showed that MT1L was related to the production of interleukin 1,6,8, and 17. It is known that interleukins are critical factors in regulating immune cells. In addition, the results revealed that MT1L plays a role in regulating immune cells such as T cells, B cells, macrophages, and lymphocytes, consistent with previous results. In addition, the CTLA4 pathway was enriched. Collectively, these results reconfirmed that MT1L probably plays an integral correlative role in tumor immunity. Another important finding was that MT1L is closely related to bladder cancer and is involved in its tumorigenesis, invasiveness, and recurrence, which suggests a potentially crucial biological functional role of MT1L in BLCA.

**Table 4:** The critical biological processes and relevant signaling pathways affected by the expression level of MT1L were predicted by GSEA. Gene sets with NES>1.5 and *p*-value < 0.05 were considered significant

Name	Enrichment scores	NES	***p***
GO_CHRONIC_INFLAMMATORY_RESPONSE	0.667	1.651	0.009
GO_INTERLEUKIN_17_PRODUCTION	0.631	1.682	0.014
GO_INTERLEUKIN_6_PRODUCTION	0.523	1.578	0.037
GO_INTERLEUKIN_1_PRODUCTION	0.511	1.547	0.05
GO_INTERLEUKIN_8_PRODUCTION	0.555	1.539	0.035
GO_MACROPHAGE_CHEMOTAXIS	0.607	1.662	0.03
GO_MACROPHAGE_DIFFERENTIATION	0.576	1.558	0.04
GO_REGULATION_OF_MACROPHAGE_CHEMOTAXIS	0.629	1.656	0.025
GO_REGULATION_OF_MONONUCLEAR_CELL_MIGRACTION	0.641	1.647	0.025
GO_REGULATION_OF_B_CELL_MEDIATED_IMMUNITY	0.629	1.585	0.039
GO_POSITIVE_REGULATION_OF_T_CELL_PROLIFERATION	0.619	1.671	0.045
GO_REGULATION_OF_LYMPHOCYTE_MIGRATION	0.569	1.598	0.03
BIOCARTA_CTLA4_PATHWAY	0.779	1.605	0.034
LINDGREN_BLADDER_CANCER_HIGH_RECURRENCE	0.67	1.749	0.014
LINDGREN_BLADDER_CANCER_CLUSTER_2B	0.661	1.862	0
OSMAN_BLADDER_CANCER_UP	0.57	1.645	0.025
ANASTASSIOU_MULTICANCER_INVASIVENESS_SIGNATURE	0.784	1.718	0.012
WANG_TUMOR_INVASIVENESS_UP	0.533	1.666	0.01
CROMER_TUMORIGENESIS_UP	0.645	1.665	0.031

## Discussion

MT1L is a pseudogene expressed in tissues of multiple cancers, including those of the brain, breast, thyroid, pancreas, etc. [29]. It has been confirmed that MT1L can be used as an indicator of prognosis in colorectal cancer [30]. However, there is limited literature on the potential biological impact of MT1L in BLCA; thus, we conducted the first analysis of this relationship.

Here, we observed that the expression of MT1L in BLCA was lower than that in normal bladder tissue using GEPIA and TIMER. GEPIA is based on TCGA and GTEX databases, while TIMER is based on TCGA, and the analysis and test methods of the two are different, so we choose two online tools for mutual verification. Logistic regression revealed that high MT1L expression was related to high T and N stage and high differentiation grade. Therefore, we reasonably hypothesized that MT1L is related to the malignant biological behavior of tumors. To verify this hypothesis, we analyzed the data from TCGA by UALCAN and obtained the correlation of MT1L expression with the OS of BLCA patients using the Kaplan-Meier plotter. The results showed that the high expression of MT1L was significantly associated with the shorter overall survival time of BLCA patients (*p*<0.05). Multivariate Cox analysis showed that MT1L expression could be an independent prognostic factor in BLCA.

The most promising finding was that in BLCA, diverse immune cell surface marker and immune infiltration levels are associated with MT1L expression. Recently, ICT has changed the intervention measures for urinary cancer (including advanced bladder cancer) [31, 32]. ICT has achieved an excellent curative effect in BLCA; however, it is undeniable that only some BLCA patients can benefit from ICT. As BLCA is a highly immunogenic malignant tumor, dysregulation of the immune response in the TME plays a decisive role in tumor occurrence and development; thus, there are still many problems to be solved regarding immunotherapy for BLCA. It is an urgent need to identify useful therapeutic targets that affect the level of immune cell infiltration. So we use TIMER and find that MT1L is related to immune infiltration in BLCA. Considering that TISIDB integrates five databases and has different analyses and test methods compared to TIMER, we use TISIDB to verify the results comprehensively. By using the TISIDB database, results indicated a correlation between MT1L expression and the infiltration of TILs. Among the 30 kinds of tumors evaluated, the correlation between MT1L expression and TILs in BLCA was represented by the deepest red color, which indicates the strongest correlation. The same result was found for the correlations between the expression level of MT1L and those of immunoregulatory molecules.

In particular, BLCA is characterized by marked infiltration by immune cells such as Tregs and MDSCs [33, 34]. Tregs and MDSCs are vital regulators of antitumor responses in BLCA, and the intratumoral presence of these cells is correlated with the poor clinical outcome that has already been established [35–37]. Tregs act as a vital immunosuppressive factor to promote tumor occurrence and development, inhibit antitumor immunity, and facilitate tumor immune escape [38]. Similarly, MDSCs are widely distributed and have strong immunosuppressive activity in the TME that inhibits cytotoxic T cell proliferation and activation, leading to the failure of the antitumor immune response and promoting cancer progression and chemoresistance [39]. TISIDB analysis indicated a substantial positive connection of MT1L expression with the infiltration levels of Tregs (*ρ*=0.708) and MDSCs (*ρ*=0.664) in BLCA. Therefore, an important conclusion can be drawn that high MT1L expression is associated with high immune infiltration levels in BLCA. Thus, we suggest that MT1L has a potential influence on tumor immunology and plays a crucial role in regulating tumor immunity.

Similarly, the relationships between the expression levels of immune cell surface markers and MT1L imply the importance of MT1L in regulating the tumor immune microenvironment in BLCA. According to TIMER, the expression of MT1L was positively correlated with that of T cell depletion markers (PD-1 and CTLA4). Under normal conditions, the interaction of PD-L1 with PD-1 can inhibit the proliferation of CD8+ T cells and the cytotoxic function mediated by T cell receptors (TCRs), thus preventing autoimmunity and resolving inflammation [31, 40]. The interaction of PD-L1 with the T cell surface receptor PD-1 leads to T cell dysfunction, which contributes to T cell failure [41]. In BLCA, high expression of MT1L was positively correlated with a high infiltration level of immune cells; moreover, it indicated poor survival and prognosis. These findings seem contradictory. However, the relationship between the expression of MT1L and that of T cell depletion markers provides a reasonable explanation for this finding.

Also, the expression of MT1L was correlated with that of the markers of tumor-associated macrophages (TAMs) and M2 macrophages, especially the cell surface markers of M2-type macrophages. TAMs are an essential component of the TME; play a vital role in the occurrence and development of tumors by mediating immunosuppression; promote tumor proliferation, metastasis, and angiogenesis; and even mediate tumor drug resistance [42–44]. TAMs can differentiate into M1 macrophages or M2 macrophages. Clinical research has shown that the poor prognosis of cancer patients significantly correlate with the number of M2 macrophages [45, 46]. The correlation between MT1L expression and M2 macrophage surface marker expression in our study suggested that MT1L may be involved in macrophage polarization and partially explains the potential mechanism of poor prognosis in BLCA patients with high expression of MT1L. Therefore, targeting MT1L may reduce the infiltration of TAMs, especially M2 macrophages. Collectively, this evidence defines the function of MT1L as a novel biomarker for the regulation of immune cell infiltration, which could provide new insight into immunotherapy.

Another meaningful finding of this study was the correlation between MT1L and interleukins shown in our GSEA. Interleukin (IL)-17 is produced by a subset of T helper (Th) cells with a strong effect on immune cells and plays an essential role in cancer pathogenesis by directly or indirectly interacting with tumors [47]. Studies have confirmed that the change patterns of IL-17 expression might be associated with the infiltration of inflammatory cells, which might also contribute to the occurrence and development of BLCA [48–50]. A reduced IL-17 level can be used as an indicator for monitoring the course of and immune response to BLCA [51]. As our results revealed, MT1L expression was closely related to interleukins, which may contribute to the likely integral correlative role of MT1L in the TME. However, the role of MT1L in other tumors needs further study.

## Conclusion

In this study, we performed a series of analyses to evaluate the role of MT1L in the tumor immune response and assess its prognostic value in BLCA. It is reasonable to conclude that high MT1L expression correlates with negative prognosis in BLCA. In addition, MT1L expression is associated with the infiltration level of different immune cells, such as T cells, Tregs, and MDSCs. Consequently, MT1L has a vital influence on immune infiltration and can be used as an independent prognostic indicator in BLCA. We hope to provide an additional feasible method for evaluating prognosis and a valuable new target for antitumor immunotherapy in BLCA. However, further research on MT1L in BLCA needs to be done.

## Data Availability

This was not applicable to this manuscript.

## Abbreviations

BLCA: Bladder cancer
MDSCs: Myeloid-derived suppressor cells
Tregs: Regulatory T cells
TAMs: Tumor-associated macrophages
OS: Overall survival
MIBC: Muscle invasive bladder cancer
MT: Metallothioneins
BRCA: Breast invasive carcinoma
CHOL: Cholangiocarcinoma
COAD: Colon adenocarcinoma
KIRP: Kidney renal papillary cell carcinoma
LIHC: Liver hepatocellular carcinoma
LUAD: Lung adenocarcinoma
LUSC: Lung squamous cell carcinoma
READ: Rectum adenocarcinoma
STAD: Stomach adenocarcinoma
THCA: Thyroid carcinoma
TILs: Tumor-infiltrating lymphocytes
MHC: Major histocompatibility complex
TME: The tumor environment
IL: Interleukin
Th: T-helper cell

## References

[1] Butt SU, Malik L (2018). Role of immunotherapy in bladder cancer: past, present and future. Cancer Chemother Pharmacol.

[2] Antoni S, Ferlay J, Soerjomataram I, Znaor A, Jemal A, Bray F (2017). Bladder cancer incidence and mortality: a global overview and recent trends. Eur Urol.

[3] Kaufman DS, Shipley WU, Feldman AS (2009). Bladder cancer. Lancet.

[4] Nadal R, Bellmunt J (2019). Management of metastatic bladder cancer. Cancer Treat Rev.

[5] Rouanne M, Roumiguié M, Houédé N, Masson-Lecomte A, Colin P, Pignot G, et al. Development of immunotherapy in bladder cancer: present and future on targeting PD(L)1 and CTLA-4 pathways. World J Urol. 2018;36(11):1727–40. 10.1007/s00345-018-2332-5. 10.1007/s00345-018-2332-5 29855698

[6] Cherian MG, Jayasurya A, Bay BH (2003). Metallothioneins in human tumors and potential roles in carcinogenesis. Mutat Res.

[7] Arriaga JM, Levy EM, Bravo AI, Bayo SM, Amat M, Aris M, et al. Metallothionein expression in colorectal cancer: relevance of different isoforms for tumor progression and patient survival. Hum Pathol. 2012;43(2):197–208. 10.1016/j.humpath.2011.04.015. 10.1016/j.humpath.2011.04.015 21820154

[8] Ruttkay-Nedecky B, Nejdl L, Gumulec J, Zitka O, Masarik M, Eckschlager T, et al. The role of metallothionein in oxidative stress. Int J Mol Sci. 2013;14(3):6044–66. 10.3390/ijms14036044. 10.3390/ijms14036044 PMC3634463 23502468

[9] Thirumoorthy N, Shyam Sunder A, Manisenthil Kumar K, Senthil Kumar M, Ganesh G, Chatterjee M (2011). A review of metallothionein isoforms and their role in pathophysiology. World J Surg Oncol.

[10] Krizkova S, Kepinska M, Emri G, Eckschlager T, Stiborova M, Pokorna P, et al. An insight into the complex roles of metallothioneins in malignant diseases with emphasis on (sub)isoforms/isoforms and epigenetics phenomena. Pharmacol Ther. 2018;183:90–117. 10.1016/j.pharmthera.2017.10.004. 10.1016/j.pharmthera.2017.10.004 28987322

[11] Milligan MJ, Harvey E, Yu A, Morgan AL, Smith DL, Zhang E, et al. Global intersection of long non-coding RNAs with processed and unprocessed pseudogenes in the human genome. Front Genet. 2016;7:26. 10.3389/fgene.2016.00026 PMC4805607 27047535

[12] Xiao-Jie L, Ai-Mei G, Li-Juan J, Jiang X (2015). Pseudogene in cancer: real functions and promising signature. J Med Genet.

[13] Yue C, Ren Y, Ge H, Yan L, Xu Y, Wang G, et al. Pseudogene DUXAP10 can be used as a diagnostic and prognostic biomarker in human cancers. J Cell Physiol. 2019;234(12):23685–94. 10.1002/jcp.28937. 10.1002/jcp.28937 31169303

[14] Yue C, Liang C, Ge H, Yan L, Xu Y, Li G, et al. Pseudogene DUXAP10 acts as a diagnostic and prognostic marker and promotes cell proliferation by activating PI3K/AKT pathway in hepatocellular carcinoma. Onco Targets Ther. 2019;12:4555–66. 10.2147/OTT.S210623. 10.2147/OTT.S210623 PMC6572670 31354289

[15] Lv W, Wang L, Lu J, Mu J, Liu Y, Dong P (2015). Downregulation of TPTE2P1 inhibits migration and invasion of gallbladder cancer cells. Chem Biol Drug Des.

[16] Zheng L, Li X, Gu Y, Ma Y, Xi T (2014). Pseudogene CYP4Z2P 3'UTR promotes angiogenesis in breast cancer. Biochem Biophys Res Commun.

[17] Mei D, Song H, Wang K, Lou Y, Sun W, Liu Z, et al. Up-regulation of SUMO1 pseudogene 3 (SUMO1P3) in gastric cancer and its clinical association. Med Oncol. 2013;30(4):709. 10.1007/s12032-013-0709-2. 10.1007/s12032-013-0709-2 23996296

[18] Blum A, Wang P, Zenklusen JC (2018). SnapShot: TCGA-analyzed tumors. Cell.

[19] Tang Z, Li C, Kang B, Gao G, Li C, Zhang Z (2017). GEPIA: a web server for cancer and normal gene expression profiling and interactive analyses. Nucleic Acids Res.

[20] Li B, Severson E, Pignon JC, Zhao H, Li T, Novak J, et al. Comprehensive analyses of tumor immunity: implications for cancer immunotherapy. Genome Biol. 2016;17(1):174. 10.1186/s13059-016-1028-7. 10.1186/s13059-016-1028-7 PMC4993001 27549193

[21] Li T, Fan J, Wang B, Traugh N, Chen Q, Liu JS, et al. TIMER: a web server for comprehensive analysis of tumor-infiltrating immune cells. Cancer Res. 2017;77(21):e108–10. 10.1158/0008-5472.CAN-17-0307. 10.1158/0008-5472.CAN-17-0307 PMC6042652 29092952

[22] Ru B, Wong CN, Tong Y, Zhong JY, Zhong SSW, Wu WC, et al. TISIDB: an integrated repository portal for tumor-immune system interactions. Bioinformatics. 2019;35:4200–2. 10.1093/bioinformatics/btz210 30903160

[23] Chandrashekar DS, Bashel B, Balasubramanya SAH, Creighton CJ, Ponce-Rodriguez I, Chakravarthi B, et al. UALCAN: a portal for facilitating tumor subgroup gene expression and survival analyses. Neoplasia. 2017;19(8):649–58. 10.1016/j.neo.2017.05.002. 10.1016/j.neo.2017.05.002 PMC5516091 28732212

[24] Gyorffy B, Lánczky A, Szállási Z (2012). Implementing an online tool for genome-wide validation of survival-associated biomarkers in ovarian-cancer using microarray data from 1287 patients. Endocr Relat Cancer.

[25] Goel MK, Khanna P, Kishore J (2010). Understanding survival analysis: Kaplan-Meier estimate. Int J Ayurveda Res.

[26] Subramanian A, Tamayo P, Mootha VK, Mukherjee S, Ebert BL, Gillette MA, et al. Gene set enrichment analysis: a knowledge-based approach for interpreting genome-wide expression profiles. Proc Natl Acad Sci U S A. 2005;102(43):15545–50. 10.1073/pnas.0506580102. 10.1073/pnas.0506580102 PMC1239896 16199517

[27] Sawant A, Hensel JA, Chanda D, Harris BA, Siegal GP, Maheshwari A, et al. Depletion of plasmacytoid dendritic cells inhibits tumor growth and prevents bone metastasis of breast cancer cells. J Immunol. 2012;189(9):4258–65. 10.4049/jimmunol.1101855. 10.4049/jimmunol.1101855 PMC3531993 23018462

[28] Facciabene A, Motz GT, Coukos G (2012). T-regulatory cells: key players in tumor immune escape and angiogenesis. Cancer Res.

[29] Holloway AF, Stennard FA, West AK (1997). Human metallothionein gene MT1L mRNA is present in several human tissues but is unlikely to produce a metallothionein protein. FEBS Lett.

[30] Hung KC, Huang TC, Cheng CH, Cheng YW, Lin DY, Fan JJ, et al. The expression profile and prognostic significance of metallothionein genes in colorectal cancer. Int J Mol Sci. 2019;20(16):3849. 10.3390/ijms20163849. 10.3390/ijms20163849 PMC6721156 31394742

[31] Wu Z, Liu J, Dai R, Wu S (2020). Current status and future perspectives of immunotherapy in bladder cancer treatment. Sci China Life Sci.

[32] Rey-Cárdenas M, Guerrero-Ramos F, Gómez de Liaño Lista A, Carretero-González A, Bote H, Herrera-Juárez M, et al. Recent advances in neoadjuvant immunotherapy for urothelial bladder cancer: what to expect in the near future. Cancer Treat Rev. 2021;93:102142. 10.1016/j.ctrv.2020.102142. 10.1016/j.ctrv.2020.102142 33453566

[33] Eruslanov E, Neuberger M, Daurkin I, Perrin GQ, Algood C, Dahm P, et al. Circulating and tumor-infiltrating myeloid cell subsets in patients with bladder cancer. Int J Cancer. 2012;130(5):1109–19. 10.1002/ijc.26123. 10.1002/ijc.26123 21480223

[34] Sjödahl G, Lövgren K, Lauss M, Chebil G, Patschan O, Gudjonsson S, et al. Infiltration of CD3^+^ and CD68^+^ cells in bladder cancer is subtype specific and affects the outcome of patients with muscle-invasive tumors. Urol Oncol. 2014;32(6):791–7. 10.1016/j.urolonc.2014.02.007. 10.1016/j.urolonc.2014.02.007 24794251

[35] Yang G, Shen W, Zhang Y, Liu M, Zhang L, Liu Q, et al. Accumulation of myeloid-derived suppressor cells (MDSCs) induced by low levels of IL-6 correlates with poor prognosis in bladder cancer. Oncotarget. 2017;8(24):38378–88. 10.18632/oncotarget.16386. 10.18632/oncotarget.16386 PMC5503539 28418913

[36] Schneider AK, Chevalier MF, Derré L (2019). The multifaceted immune regulation of bladder cancer. Nat Rev Urol.

[37] Chen Z, Zhou L, Liu L, Hou Y, Xiong M, Yang Y, et al. Single-cell RNA sequencing highlights the role of inflammatory cancer-associated fibroblasts in bladder urothelial carcinoma. Nat Commun. 2020;11(1):5077. 10.1038/s41467-020-18916-5. 10.1038/s41467-020-18916-5 PMC7545162 33033240

[38] Li C, Jiang P, Wei S, Xu X, Wang J (2020). Regulatory T cells in tumor microenvironment: new mechanisms, potential therapeutic strategies and future prospects. Mol Cancer.

[39] Groth C, Hu X, Weber R, Fleming V, Altevogt P, Utikal J, et al. Immunosuppression mediated by myeloid-derived suppressor cells (MDSCs) during tumour progression. Br J Cancer. 2019;120(1):16–25. 10.1038/s41416-018-0333-1. 10.1038/s41416-018-0333-1 PMC6325125 30413826

[40] Yang R, Sun L, Li C-F, Wang Y-H, Yao J, Li H, et al. Galectin-9 interacts with PD-1 and TIM-3 to regulate T cell death and is a target for cancer immunotherapy. Nat Commun. 2021;12(1):832. 10.1038/s41467-021-21099-2. 10.1038/s41467-021-21099-2 PMC7864927 33547304

[41] Alsaab HO, Sau S, Alzhrani R, Tatiparti K, Bhise K, Kashaw SK, et al. PD-1 and PD-L1 checkpoint signaling inhibition for cancer immunotherapy: mechanism, combinations, and clinical outcome. Front Pharmacol. 2017;8:561. 10.3389/fphar.2017.00561. 10.3389/fphar.2017.00561 PMC5572324 28878676

[42] Pinton L, Masetto E, Vettore M, Solito S, Magri S, D’Andolfi M, et al. The immune suppressive microenvironment of human gliomas depends on the accumulation of bone marrow-derived macrophages in the center of the lesion. J ImmunoTher Cancer. 2019;7(1):58. 10.1186/s40425-019-0536-x. 10.1186/s40425-019-0536-x PMC6391795 30813960

[43] Qian B-Z, Pollard JW (2010). Macrophage diversity enhances tumor progression and metastasis. Cell.

[44] Komohara Y, Takeya M (2017). CAFs and TAMs: maestros of the tumour microenvironment. J Pathol.

[45] Murray Peter J, Allen Judith E, Biswas Subhra K, Fisher Edward A, Gilroy Derek W, Goerdt S, et al. Macrophage activation and polarization: nomenclature and experimental guidelines. Immunity. 2014;41(1):14–20. 10.1016/j.immuni.2014.06.008. 10.1016/j.immuni.2014.06.008 PMC4123412 25035950

[46] Chen H, Yao J, Bao R, Dong Y, Zhang T, Du Y, et al. Cross-talk of four types of RNA modification writers defines tumor microenvironment and pharmacogenomic landscape in colorectal cancer. Mol Cancer. 2021;20(1):29. 10.1186/s12943-021-01322-w. 10.1186/s12943-021-01322-w PMC7869236 33557837

[47] Wu H-H, Tsai L-H, Huang C-K, Hsu P-H, Chen M-Y, Chen Y-I, et al. Characterization of initial key steps of IL-17 receptor B oncogenic signaling for targeted therapy of pancreatic cancer. Sci Transl Med. 2021;13(583):eabc2823. 10.1126/scitranslmed.abc2823. 10.1126/scitranslmed.abc2823 33658352

[48] Liu Y, Yang W, Zhao L, Liang Z, Shen W, Hou Q, et al. Immune analysis of expression of IL-17 relative ligands and their receptors in bladder cancer: comparison with polyp and cystitis. BMC Immunol. 2016;17(1):36. 10.1186/s12865-016-0174-8. 10.1186/s12865-016-0174-8 PMC5048669 27716046

[49] Wang L, Yi T, Kortylewski M, Pardoll DM, Zeng D, Yu H (2009). IL-17 can promote tumor growth through an IL-6-Stat3 signaling pathway. J Exp Med.

[50] Dowell AC, Cobby E, Wen K, Devall AJ, During V, Anderson J, et al. Interleukin-17-positive mast cells influence outcomes from BCG for patients with CIS: data from a comprehensive characterisation of the immune microenvironment of urothelial bladder cancer. PLoS One. 2017;12(9):e0184841. 10.1371/journal.pone.0184841. 10.1371/journal.pone.0184841 PMC5607173 28931051

[51] Baharlou R, Ahmadi Vasmehjani A, Dehghani A, Ghobadifar MA, Khoubyari M (2014). Reduced interleukin-17 and transforming growth factor Beta levels in peripheral blood as indicators for following the course of bladder cancer. Immune Netw.

